# Angiotensin II, Aldosterone, and Anti-Inflammatory Lymphocytes: Interplay and Therapeutic Opportunities

**DOI:** 10.1155/2012/829786

**Published:** 2012-05-21

**Authors:** Daniel Arthur B. Kasal, Ernesto L. Schiffrin

**Affiliations:** ^1^Departamento de Clínica Médica, Faculdade de Ciências Médicas, Universidade do Estado do Rio de Janeiro, Avenida. 28 de Setembro 77, 3^o^.andar, Sala 329, Vila Isabel, 20551-030 Rio de Janeiro, RJ, Brazil; ^2^Department of Medicine and Lady Davis Institute for Medical Research, Sir Mortimer B. Davis-Jewish General Hospital, McGill University, Montreal, QC, Canada H3T 1E2

## Abstract

Inflammation is recognized as an important factor in the pathophysiology of hypertension, with the renin-angiotensin-aldosterone system (RAAS) playing a key role in the disease. Initially described because of its contribution to extracellular fluid and electrolyte homeostasis, the RAAS has been implicated in endothelial dysfunction, vascular remodeling, oxidative stress, proinflammatory cytokine production, and adhesion molecule synthesis by the vascular wall. Both angiotensin II and aldosterone are involved in these systemic effects, activating innate and adaptive immune responses. This paper highlights some aspects connecting RAAS to the hypertensive phenotype, based on experimental and clinical studies, with emphasis on new findings regarding the contribution of an increasingly studied population of T lymphocytes: the T-regulatory lymphocytes. These cells can suppress inflammation and may exert beneficial vascular effects in animal models of hypertension.

## 1. Introduction

The major impact of hypertension on the population is well recognized by health care providers and to some degree by the general public. Data from the International Hypertension Society estimate that hypertension is associated with approximately half of deaths caused by cardiovascular disease, representing around eight million deaths per year around the world [1]. Notwithstanding the importance on health systems, the determinants of hypertension remain obscure in the majority of patients seen on routine clinical practice, who accordingly are diagnosed as having essential or primary hypertension. This term was coined almost one century ago, at a time when cell and molecular biology were just beginning to appear as disciplines. Indeed, among the first reports of essential hypertension there is the paper by L. M. Brown, who wrote in 1929: *“I am presenting this type of hypertension as a definite clinical entity, separate from the high tension associated with diseases of the heart, kidney and hyperthyroidism*” [2]. One decade later, other authors suggested that research on arterial hypertension would soon lead to the replacement of the rather unspecific essential hypertension by a number of hypertensive syndromes with defined and distinct pathophysiologic pathways [3].

More than sixty years have passed since these seminal studies, and still most patients are diagnosed as having essential hypertension. Nevertheless, we know much more about the mechanisms involved in the genesis and progression of the disease. The role of mediators of the renin-angiotensin-aldosterone system (RAAS), the contribution of genetic polymorphisms, endothelial dysfunction, and oxidative stress, among others, are features that evolve in parallel and interact with each other, resulting in the hypertensive phenotype.

In this paper we will focus on phenomena concerning two vital systems which are deep-rooted in evolution and are present in every vertebrate: the immune response and RAAS. Both offer the ability to cope with challenges imposed by the environment, whether the exposure to an antigen (as in inflammation), or a shift in water and sodium balance (as is the case of RAAS), allowing the organism to keep volume homeostasis despite wide variations in water and sodium intake. We will also comment on oxidative stress, one of the main mechanisms by which RAAS exerts its proinflammatory actions in the vessel wall or the kidney. Finally, we will deal with a special group of immune cells, the regulatory T lymphocytes. This lymphocyte population acts by suppressing inflammation and has been the object of exciting recent studies about the interplay between blood pressure, immune response, and RAAS.

## 2. The Renin-Angiotensin-Aldosterone System and Inflammation

By far the best known properties of the RAAS have been, since the first description of renin in 1898 [4], linked to its hemodynamic and pressor effects. Accordingly, fifty years ago many aspects were known about the actions of RAAS on kidney sodium and water reabsorption, as well as the vasoconstrictor effects of angiotensin (Ang) II. The first reports associating inflammation and high blood pressure appeared at this time [5]. Nevertheless, studies establishing the connection between the RAAS and the immunologic response would be published only in the following decades.

In one of the first works of immune involvement in hypertension, Rodriguez-Iturbe et al. demonstrated that chronic Ang II infusion caused renal infiltration of T lymphocytes in rats. This effect was blunted by the treatment with the immunosuppressor mycophenolate mofetil, whose actions were independent of arterial pressure [6]. Further studies have shown that both Ang II and aldosterone, in association with inflammatory mediators such as interferon-*γ* (IFN-*γ*) and tumour necrosis factor *α* (TNF-*α*), are able to stimulate growth and proliferation of vascular smooth muscle cells (VSMCs), leading to vascular hypertrophy characteristic of hypertension [7].

In another set of studies linking RAAS and inflammation, the contribution of macrophages in Ang II-induced vascular lesions was evaluated in animals with impairment of innate immunity, the osteopetrotic (Op) mice [8]. These animals display macrophage deficiency due to a mutation of macrophage colony-stimulating factor (*mCSF*) gene. Op mice did not develop hypertension, endothelial dysfunction, and vascular remodelling when subjected to chronic Ang II infusion, when compared to control. The role of monocytes in Ang II-induced vascular effects was further demonstrated by Wenzel et al. [9]. In transgenic mice (LysM^iDTR^) subjected to conditional depletion of myelomonocytic cells, there was a reduction in Ang II-induced hypertension, vascular dysfunction, and oxidative stress. Reconstitution of depleted mice with the adoptive transfer of monocytes, but not neutrophils, reestablished the aforementioned features.

The association of adaptive immunity in Ang II-induced hypertension was also studied by Shao et al., who showed that Ang II infusion in rats triggered lymphocyte recruitment to the kidney [10]. This effect was prevented by the angiotensin type I receptor blocker olmesartan, but not by the vasodilator hydralazine. The importance of T lymphocytes in the genesis of vascular lesions induced by Ang II was shown in mice by Guzik et al. [11]. Using animals lacking T and B lymphocytes (rag-1^−/−^), the authors demonstrated that hypertension, endothelial dysfunction, vascular remodelling, and superoxide production induced by Ang II were reduced in rag-1^−/−^ mice and restored by T-lymphocyte adoptive transfer, but not when B lymphocytes were used. In addition, the same paper showed that treatment with etanercept, a TNF-*α* inhibitor, prevented Ang II-induced hypertension and superoxide generation.

Ang II can modulate adaptive immunity, acting directly on lymphocytes. Both T and B lymphocytes express angiotensin type 1a receptors (AT_1a_R) in mice, and *in vitro*, Ang II stimulates the proliferation of splenic lymphocytes [12]. These findings, added to evidences that Ang II and its precursors, angiotensinogen and Ang I, are capable of inducing human T lymphocyte and Natural Killer cell (NK) proliferation [13], have suggested the presence of an intracellular RAAS. In addition, human T lymphocytes express renin and its receptors, angiotensinogen, angiotensin I-converting enzyme (ACE), and angiotensin II receptors type I and II. In a similar way, mouse T lymphocytes express a local RAAS, regulating lymphocyte activation, tissue homing markers, and the production of TNF-*α* [14].

A new mechanism linking inflammation and high blood pressure mediated by Ang II was proposed by Marvar et al. Using mice subjected to a lesion in the anteroventral region of the third cerebral ventricle and infused with Ang II for 2 weeks, these authors observed a blunting of Ang II pressor effects, vascular oxidative stress, circulating T-lymphocyte activation, and their vascular infiltration [15]. In a subset of experiments in the same study, hydralazine blunted Ang II-induced hypertension, and this was associated with a reduction in lymphocyte activation. However, there was no evidence of a direct hydralazine action on the capacity of lymphocytes to display antigen-specific activation. The authors suggested that Ang II effects on the central nervous system caused an elevation of blood pressure that could in turn activate T lymphocytes and vascular inflammation.

Within the RAAS, aldosterone is the mediator stimulated by Ang II and contributes to the sequence of events leading to hypertension. There is abundant evidence linking aldosterone to target organ lesions, in association with oxidative stress and inflammation. In experimental models of hypertension, treatment with the mineralocorticoid receptor (MR) blocker spironolactone was able to reduce cerebral and renal vascular lesions, cardiac hypertrophy, inflammation, and extracellular matrix synthesis [16]. Rocha et al. have shown that aldosterone infusion for 4 weeks, associated with an increase in sodium intake, produced extended arterial inflammatory lesions, with myocardial perivascular macrophage deposition [17]. The selective MR blocker eplerenone reduced this inflammatory response. The beneficial effects of this drug were also verified in the peripheral vasculature, with reduction of inflammatory cell infiltration, fibrosis, and aortic hypertrophy in hypertensive rats [18]. An interesting interplay between Ang II and aldosterone was described by Virdis et al. In rats chronically infused with Ang II, spironolactone treatment blunted Ang II-induced endothelial dysfunction, resistance artery remodeling, and aortic redox state [19]. These findings underscore that vascular damage caused by Ang II is mediated, at least in part, via stimulation by aldosterone of the MR receptor.

Both human and experimental model researches have shown that aldosterone can act directly on vessel wall components and inflammatory cells. Human VSMCs exposed to aldosterone present an increase in type I and III collagen, interleukin- (IL-) 16, and cytotoxic T-lymphocyte-associated protein 4 expression, molecules associated with fibrosis, inflammation, and vascular calcification [20]. Macrophages possess MR and its expression increases in response to INF-*γ*, secreted by T lymphocytes [21]. In addition, Leibovitz et al. demonstrated that in Op mice, chronic aldosterone infusion does not induce endothelial dysfunction and vascular cell adhesion molecule (VCAM-1) expression, providing additional evidence for the role of inflammatory cells and specifically macrophages in aldosterone-induced vascular damage [22].

## 3. Inflammation and Oxidative Stress

Studies on immunity and hypertension show a close relationship between inflammatory cell infiltration and oxidative stress in cardiovascular tissues. Indeed, one of the main mechanisms by which RAAS causes vascular pathology in hypertension involves reactive oxygen species (ROS) production. Superoxide (^∙^O_2_^−^), hydroxyl radical (OH^−^) and hydrogen peroxide (H_2_O_2_), and lipid peroxidation unstable products belong to this group of chemically reactive compounds [23]. Free radicals are able to interact with virtually all biologic molecules, including lipids, proteins, nucleic acids, carbohydrates, and nitric oxide (NO). They are involved in cell growth and proliferation as well as extracellular matrix expansion. The consequences of ROS production on the cardiovascular system are cell injury and endothelial dysfunction, since free radicals inactivate NO, transforming it into peroxynitrite, which leads to impaired vasodilation [24].

 Studies performed in the last decade have helped elucidate mechanisms whereby the RAAS causes ROS elevation. Both Ang II and aldosterone induce the expression of reduced nicotinamide adenine dinucleotide phosphate (NADPH) oxidase, the main enzyme responsible for the production of superoxide in vascular tissue [26]. Free radicals, in turn, act as activators of inflammation. Oxidative stress triggers an inflammatory process by stimulating vascular permeability, increasing the secretion of mediators such as prostaglandins and vascular endothelial growth factor (VEGF) [27]. The next steps, represented by adhesion and diapedesis of inflammatory cells into the vasculature, are also governed by ROS production. Ang II increases the expression of cell adhesion molecules VCAM-1, intercellular cell adhesion molecule 1 (ICAM-1), and E-selectin through signaling pathways involving ROS production. This phenomenon is amplified by vessel wall invasion by inflammatory cells, which are rich in NADPH oxidase and enhance local oxidative stress [28]. At the end of the process, tissue repair mechanisms are also affected by oxidative stress. Both Ang II and aldosterone stimulate hyperplasia, hypertrophy, and apoptosis, as well as vascular fibrosis [29]. The resulting cell proliferation and matrix deposition, mainly collagen and fibronectin, produce vascular remodelling and increased vascular stiffness. Taking into account the aforementioned features leading to target organ lesion, therapeutic interventions aiming to modulate vascular redox state, as well as immunological activation, could reduce hypertension morbidity.

## 4. T-Regulatory Lymphocyte and Hypertension

A specific subset of T lymphocytes has recently become the focus of studies on inflammation-linked vascular lesions. T-regulatory lymphocytes (Treg) can suppress inflammatory actions of other lymphocytes, as well as macrophages, dendritic cells and neutrophils [30]. Initially evaluated in the context of autoimmune diseases, graft rejection, and malignancies, Treg properties are increasingly recognized in cardiovascular disease.

The first reports of a population of CD4^+^ T cells able to suppress immunological reactions were published more than 20 years ago. One of the seminal studies used a model of lymphocyte infusion in rats, which could reduce host rejection to cardiac transplantation [31]. Shortly after, the characterization of a subset of CD4^+^CD25^+^ T cells able to suppress innate and adaptive immune responses was accomplished. An important advance in the knowledge of these cells was the description of patients bearing the IPEX syndrome (immune dysregulation, polyendocrinopathy, enteropathy, X-linked), a fatal disease characterized by the development of autoimmune disorders in the first years of life due to a mutation in the transcription factor forkhead box protein 3 (Foxp3) [32]. Other studies confirmed Foxp3 as the transcription factor necessary for the maturation of CD4^+^ T lymphocytes into Treg. A reduction in Treg is associated with the development of autoimmune diseases in both humans and experimental animals [33].

 There are several mechanisms that have been proposed through which Tregs suppress proinflammatory actions of other T-lymphocyte subtypes. The Treg surface marker CD25 is a receptor of IL-2, a cytokine produced by T-effector cells and amplifier of the inflammatory response, promoting Th1-associated gene expression in immature thymocytes [34]. Following CD25 binding, IL-2 is internalized and degraded, hence reducing its bioavailability [35]. Treg can also secrete inhibitory cytokines IL-35 and IL-10 [36], which produce cell cycle arrest, leading to an interruption of inflammatory cell clonal expansion. Alternative mechanisms for Treg effects include lysis of target cells through the secretion of granzyme B and the expression of galectin-1 [37], causing a blockade in the production of proinflammatory cytokines by other T lymphocytes and their apoptosis.

**Figure 1:**
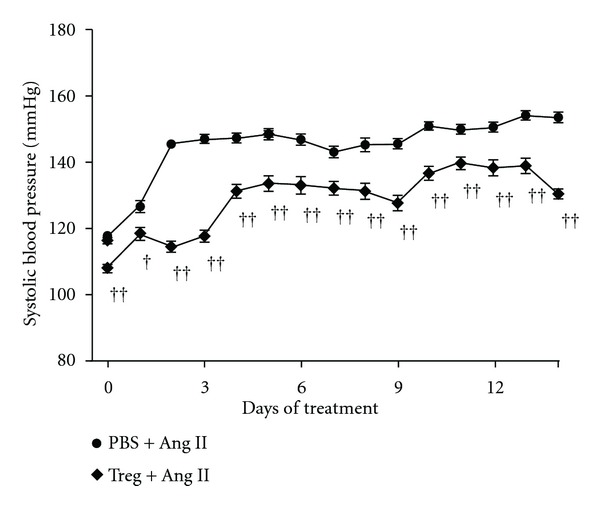
T-regulatory lymphocyte (Treg) adoptive transfer prevented angiotensin II (Ang II)-induced hypertension. Systolic blood pressure (SBP) was evaluated by telemetry in mice chronically infused with Ang II and pretreated with PBS or Treg. Mean daily SBP data are presented. Data are expressed as means ± SEM. ^†^ *P* < 0,05 e ^††^ *P* < 0,001 versus PBS + Ang II with *n* = 24 data points per day for each 3 to 4 mice. Adapted from [25].

Recently, we performed studies looking at the interaction between inflammatory cells in a RAAS activation experimental model using Treg adoptive transfer. In mice receiving chronic Ang II infusion, Treg intravenous administration prevented high blood pressure, vascular oxidative stress and macrophage, and T-cell infiltration in aorta, when compared to untreated hypertensive animals [25]. The blood pressure result can be seen in Figure 1. Similar findings were observed when Tregs were administered to aldosterone-infused animals, although in this case the effect on blood pressure was negligible [38]. These results show that the RAAS proinflammatory actions on both innate and adaptive immune responses run in concert with oxidative profile and pressor effects, and all have the capacity to be modulated by interventions that target the immune system.

## 5. Conclusion

 Multiple research lines associate cardiovascular disease, including hypertension, to a low-level chronic inflammatory state. Current evidence in favour to this at least with respect to high blood pressure is predominantly based on experimental models of hypertension, although increases in C-reactive protein (a marker of systemic inflammation) in human subjects have been correlated with both incident hypertension and the level of blood pressure elevation, independent of other cardiovascular risk factors [39, 40].

The expectation expressed by hypertension researchers in the beginning of the last century that essential hypertension would be replaced by other terms with precise pathophysiological characteristics has not been fulfilled yet. Indeed, the multifactorial nature of hypertensive mechanisms makes it difficult to identify a predominant mediator in most cases. However, the vast number of studies that followed the first descriptions of essential hypertension has allowed an understanding of the contribution of new mechanisms. The effects of Ang II and aldosterone are mediated, at least in part, by the production of ROS by macrophages. Cellular and molecular immunological phenomena causing vascular damage in hypertension represent a new frontier in research that could result in an improvement of our therapeutic armamentarium for cardiovascular disease.
