# Erratum to “Angiotensin II, Aldosterone, and Anti-Inflammatory Lymphocytes: Interplay and Therapeutic Opportunities”

**DOI:** 10.1155/2012/132598

**Published:** 2012-09-03

**Authors:** Daniel Arthur B. Kasal, Ernesto L. Schiffrin

**Affiliations:** ^1^Departamento de Clínica Médica, Faculdade de Ciências Medicas, Universidade do Estado do Rio de Janeiro, Avenida. 28 de Setembro 77, 3°.andar, Sala 329, Vila Isabel, 20551-030 Rio de Janeiro, RJ, Brazil; ^2^Department of Medicine and Lady Davis Institute for Medical Research, Sir Mortimer B. Davis-Jewish General Hospital, McGill University, Montreal, QC, Canada H3T 1E2

The following corrections should be considered.

- In the text on page 3, replace the number of reference [26] by [25].
- In the text on page 3, replace the number of reference [27] by [26].
- In the text on page 3, replace the number of reference [28] by [27].
- In the text on page 3, replace the number of reference [29] by [28].
- In the text on page 3, replace the number of reference [30] by [29].
- In the text on page 3, replace the number of reference [31] by [30].
- In the text on page 3, replace the number of reference [32] by [31].
- In the text on page 3, replace the number of reference [33] by [32].
- In the text on page 3, replace the number of reference [34] by [33].
- In the text on page 3, replace the number of reference [35] by [34].
- In the text on page 3, replace the number of reference [36] by [35].
- In the text on page 3, replace the number of reference [37] by [36].
- In the text on page 4, replace the number of reference [25] by [37].
- In the legend of Figure 1, replace the number of reference [25] by [37].
- Please consider the following corrected reference list for paper.
